# Sexual Orientation-Specific Policies Are Associated With Prenatal Care Use in the First Trimester Among Sexual Minority Women: Results From a Prospective Cohort Study

**DOI:** 10.1093/abm/kaae037

**Published:** 2024-07-11

**Authors:** Bethany G Everett, Zoë Bergman, Brittany M Charlton, Veronica Barcelona

**Affiliations:** Department of Sociology, University of Utah, Salt Lake City, UT, USA; Department of Sociology, University of Utah, Salt Lake City, UT, USA; Department of Population Medicine, Harvard Medical School and Harvard Pilgrim Health Care Institute, Cambridge, MA, USA; Columbia University School of Nursing, New York, NY, USA

**Keywords:** Prenatal care, Sexual orientation, Policy, Health status disparity

## Abstract

**Background:**

Previous research has shown sexual minority women (SMW) are more likely to report multiple maternal and infant health outcomes compared to heterosexual women and that these outcomes are moderated by the policy environment. Little is known, however, about prenatal care use disparities or the social determinants of prenatal care use for SMW.

**Purpose:**

To examine the relationship between sexual orientation-specific policies that confer legal protections (e.g., hate crime protections, housing discrimination, same-sex marriage) and prenatal care use among women using a prospective, population-based data set.

**Methods:**

Using the National Longitudinal Study of Adolescent to Adult Health and logistic regression, we link measures of state policies to the use of prenatal care in the first trimester among women who had live births. The use of prospective data allows us to adjust for covariates associated with preconception care use prior to pregnancy (*n* = 586 singleton births to SMW; *n* = 4,539 singleton births to heterosexual women).

**Results:**

Sexual orientation-specific policies that conferred protections were associated with increased use of prenatal care among pregnancies reported by SMW (OR = 1.86, 95% CI 1.16, 2.96). In fact, in states with zero protections, we found no differences in prenatal care use by sexual minority status; however, in states with two or more protective policies, SMW were more likely to access prenatal care in the first trimester than heterosexual women. There was no relationship between sexual orientation-specific policy environments and prenatal care use among pregnancies reported by heterosexual women.

**Conclusions:**

Recent research has documented that SMW are more likely to have adverse perinatal and obstetrical outcomes than their heterosexual peers. These findings suggest that Lesbian/Gay/Bisexual-specific policy protections may facilitate the use of prenatal care among SMW, a potentially important pathway to improve reproductive health among this population.

## Teaser

Sexual orientation-specific legal protections are associated with increased use of prenatal care in the first trimester for pregnant sexual minority women.

## Introduction

Early and ongoing prenatal care is linked to fewer birth complications and improved maternal health outcomes [1, 2]. Access to prenatal care in the USA, however, is inconsistent; previous research has shown inequities in access and use of prenatal care by socioeconomic status, race, and ethnicity [3, 4]. One of the first steps to ensure health equity in childbirth is to improve access to quality care throughout pregnancy, delivery, and postpartum. Early prenatal care initiation is imperative to improved outcomes as it allows for more accurate dating of pregnancy, assessment, referral to social support programs, and opportunities for screening [5, 6]. A recent review of prenatal care needs and effectiveness found that pregnant study participants wanted patient-centered care that provided information about social support and economic resources and to be able to ask providers questions about their pregnancy and other forms of healthcare services [7]. The variety of desired information and resources demonstrated by pregnant patients suggests that prenatal care interactions are a unique opportunity to address multiple health concerns as well as other social determinants of health, including poverty and social isolation. Other work has suggested that prenatal care may also improve postnatal outcomes and reduce hospital length stays after delivery [8]. Unfortunately, no research has examined sexual orientation disparities in prenatal care use in the first trimester.

A growing body of work has documented sexual orientation disparities in maternal and infant health, suggesting that sexual minority women (women who identify as bisexual, gay, or lesbian, report same-sex attraction or behavior; SMW) are more likely to report poorer preconception health [9], adverse birth outcomes (e.g., preterm and low birth weight infants) [10–12], not initiating breastfeeding [13], birth complications [12], and higher rates of smoking during pregnancy [14]. One mechanism that may contribute to these outcomes is the underuse of prenatal care. Studies have shown that among the general population, SMW are less likely to be insured and less likely to use sexual and reproductive health services [15–18].

Studies focused on prenatal care use by SMW have primarily used qualitative data and clinical samples. These studies have documented that SMW face increased vulnerability, stigma, and bias in reproductive health spaces [19–22]. These experiences often lead to greater mistrust of the healthcare system among SMW, a reluctance to return to health spaces, and lower rates of disclosing their identities to medical providers [23]. In particular, women in same-sex relationships describe combatting assumptions of heterosexuality from healthcare providers and discriminatory interactions with such providers [24–27].

Increasingly, research has suggested that social and political landscapes influence healthcare use and various health outcomes among sexual minority persons [28–32]. The U.S. policy landscape for sexual minorities has experienced multiple changes over the last two decades. According to the Movement Advancement Project, which tracks lesbian, gay, bisexual, transgender or queer (LGBTQ) state-policy environments, between 2010 and 2020, the number of LGBTQ persons living in states with “negative” or hostile policy environments fell by half, indicating overall shifts toward more accepting and supportive policy environments [33]. For example, in 2010, 12 states prevented housing and employment discrimination based on sexual orientation compared to 20 states in 2020. Similarly, between 2010 and 2020, the number of states that banned discrimination based on sexual orientation in school settings and health care more than doubled. And while just 14 states recognized same-sex marriage or civil unions in 2010, in 2015, same-sex marriage became legal at the federal level for all U.S. citizens.

As of 2023, 77% of sexual minorities live in states with hate crime statutes that include sexual orientation, while 60% live in states that prohibit adoption discrimination based on sexual orientation. It is important to note, however, that even as the previous decade was primarily characterized by overall improvements in state-policy environments for sexual minorities, there have been an unprecedented number of state legislative bills introduced and passed targeted toward LGBTQ persons. The ACLU policy tracker identified 506 anti-LGBTQ bills introduced in 2023 state legislative sessions [34].

Pregnancy outcomes may be particularly sensitive to these policies since sexual minorities face additional legal challenges in childbearing and family formation (e.g., second-parent adoption). Only one study has examined the impact of sexual orientation-specific policies and birth outcomes and found that lesbian-identified women in states with more protective policies had a decreased risk of preterm birth and higher birthweight infants; these policies did not affect any other sexual identity group [35]. No research to date has examined sexual orientation disparities in prenatal care use nor the impact of state-level sexual orientation policies on the use of prenatal care in the first trimester. This study addresses this gap using a national, prospective data set that includes sexual orientation measures, several sexual orientation-specific state-level policies, and pregnancy-related health behaviors and outcomes, including prenatal care use in the first trimester. In addition, the prospective nature of the study allows us to ensure that the policy environment exposure was measured prior to the pregnancy and adjust for preconception factors related to prenatal care use. Therefore, the purpose of the present study was to examine whether state-level Lesbian/Gay/Bisexual (LGB) policy protections and sexual minority status were associated with receiving prenatal care in the first trimester of pregnancy. We hypothesized that SMW who lived in states with fewer LGB policy protections prior to their births would have lower rates of first-trimester prenatal care compared to heterosexual women and SMW who lived in states with more policy protections for LGB people prior to birth.

## Data and Measures

Data for this analysis came from the National Longitudinal Study of Adolescent to Adult Health (Add Health). The initial sample was drawn in 1994 from 80 high schools and 52 middle schools throughout the USA with an unequal probability of selection (Wave I; aged 12–18) [36]. A subsample of students (*n* = 20,745) was asked to complete additional in-home interviews and contacted for follow-up interviews between 2001 and 2002 (Wave III; aged 18–26), 2007–2008 (Wave IV; aged 24–32), and 2016–2018 (Wave V; aged 34–43). Response rates for these three waves were 77.4%, 80.3%, and 69.3%, respectively.

Respondents were asked about their pregnancy and birth histories in Waves IV and V of the study, including the date of each live birth and whether, for each birth, they received prenatal care. Additionally, a supplementary file was released by Add Health in 2020 that provided information on policies related to sexual orientation-based protections for the state in which respondents resided during Wave III and Wave IV of the survey [37]. Importantly, because data are available on the date of interview and date of live birth, we can correctly time-order exposure to sexual orientation-specific policies *prior* to all births. Our analyses were restricted to singleton live births that occurred after Wave III of survey data collection, resulting in a final sample size of 5,125 singleton births to 3,103 women.

### Measures

#### Outcome measure

Prenatal care in the first trimester was derived from a survey item that asked respondents, “During this pregnancy, did you visit a doctor, nurse-midwife, or other health care provider for prenatal care, that is, for one or more pregnancy check-ups?” and “How many weeks pregnant were you at the time of the first prenatal care visit?” We created a dichotomous variable from these two questions that captured whether a respondent had received prenatal care before 13 weeks (first trimester; 1 = yes) or not (0 = no).

#### Independent variables

*LGB policy protections* were measured using a scale of four state-level policies related to sexual orientation-based protections in (i) employment, (ii) hate crimes, (iii) same-sex marriage, and (iv) same-sex adoption [37]. Policy indicators were derived from the Human Rights Council (HRC) and the American Civil Liberties Union (ACLU). The HRC collects and disseminates information on LGBT laws and policies related to topics such as marriage, adoption, hate crimes, and employment. In addition, every year, the HRC releases a state-by-state summary of LGBT laws and policies. Three variables in this data file were generated using information from the HRC. These variables include employment discrimination, hate crime legislation, and relationship recognition. The ACLU collects and disseminates information on significant issues related to individual rights and liberties, including LGBT rights. One variable was generated using information from the ACLU: whether the state had an adoption law pertaining to sexual minorities.

Home addresses and latitude/longitude coordinates were collected using the global positioning system devices at the time of data collection. An ancillary contextual file used respondent location in Waves III and IV of survey data collection to match locations to their state of residence. Wave III measures correspond to the period following Wave II to Wave III (1997–2002). For instance, if a state implemented hate crime statutory provisions based on sexual orientation before or during 2002, then respondents are coded as having this protection at the time of the Wave III interview. Wave IV measures correspond to the period following Wave III and ending at Wave IV (2003–2008). If a state lacked a hate crime statutory provision based on sexual orientation in the years spanning 2003–2008, then respondents are classified as not having this provision at the time of the Wave IV interview, even if the state had such a provision at some point before 2003.

We combined these policies to create a scale that ranges from 0 to 3 or more policies. Using both survey dates and dates of births, we were able to assign each singleton birth the number of LGB policy protections that most closely preceded the birth from either Wave III or Wave IV of the survey. We also examined these policy variables separately to test whether there was an association between any single protective policy and prenatal care use.

*Sexual orientation identity* was measured in Waves III, IV, and V. Respondents were asked, “Please choose the description that best fits how you think about yourself: 100% heterosexual (straight); mostly heterosexual (straight), but somewhat attracted to people of your own sex; bisexual, that is, attracted to men and women equally; mostly homosexual (gay), but somewhat attracted to people of the opposite sex; 100% homosexual (gay).” Using the data from the interview and date of birth, we measured sexual identity using the identity reported before the birth. Supplementary analyses showed that the results were similar for all sexual minority groups; thus, we created a single dichotomous variable that captures whether a woman identified with any sexual minority identity (e.g., mostly heterosexual, bisexual, gay/lesbian) or as “100% heterosexual” (referent).

#### Covariates

We included a variety of covariates associated with obstetrical care utilization, including obstetrical care. These include race/ethnicity [2], maternal age [38, 39], relationship status [40, 41], education [42], birth order [43], and insurance status [44]. As an additional measure of socioeconomic status (SES), we include an indicator of childhood poverty, as research has shown childhood SES has far-reaching consequences for future SES and health behaviors [45].

*Race and ethnicity* were measured as a categorical variable capturing whether a respondent identified as Non-Hispanic White (referent), Non-Hispanic Black, Hispanic, or Other. *Maternal age* was calculated based on date of birth and date of interview at the time of the pregnancy. *Relationship status* at the time of pregnancy was defined as a dichotomous variable capturing whether the respondent was not married to, or cohabiting with, her pregnancy partner at the time of the birth (1 = yes; 0 = no). That is, for each pregnancy, relationship information that corresponds to the partner at the time of pregnancy was also collected. Because our focus is on SMW, we use the term pregnancy partner to include women who are in same-sex relationships.

*Educational attainment* was captured with a dichotomous variable indicating whether the respondent had graduated college before the pregnancy (1 = yes; 0 = no). Previous research has found that a college degree, or the equivalent of 16 years of education, is an important cutoff for prenatal care use [42]. *Adolescent poverty* was measured with a dichotomous variable capturing whether the respondent’s family income fell below the federal poverty level at Wave I of the survey. *Insurance status* before pregnancy was also defined using a categorical variable indicating whether the respondent has no insurance (referent), private insurance, Medicaid, or other insurance coverage. *Birth order* captures whether the index pregnancy is the first birth (referent), second, third, or higher birth.

### Analytic Strategy

The data were structured so births were the unit of analysis rather than individual respondents. This approach allowed each birth to be considered a unique observation and for the correct time ordering of covariates due to the longitudinal nature of the data. First, we calculated descriptive statistics for the total sample and by sexual minority status. We conducted bivariate tests to examine if study covariates varied by maternal sexual identity. Second, we used multivariable logistic models clustered on the mother’s study ID to account for multiple births occurring to a single respondent. All models included an interaction between sexual minority status and sexual orientation policies to determine if the relationship between LGB policies varied by sexual orientation. Third, we conducted a series of additional analyses to disaggregate the policy scale into single policy indicators to determine the unique contribution of each policy indicator for prenatal care. The figure was created using the “margins” command in Stata 16.0. To make Add Health’s estimates nationally representative and to account for the data’s complex sampling frame, all models adjusted for Add Health’s design structure using the population weight [46].

## Results

### Descriptive Analyses

In Table 1 we provide descriptive statistics for the total sample of births and by sexual minority status. Overall, most women received prenatal care in their first trimester (94%); no differences were observed by sexual minority status in prenatal care use. The total sample was mostly white (71.7%) and had a mean maternal age of 28. This means maternal age was slightly elevated as pregnancies that ended before Wave III were not eligible for this sample. Just 13.7% of the sample was unmarried or cohabitating with their pregnancy partner, and 28.6% had a college degree before pregnancy. Most births were to privately insured women (67.8%), but almost 20% were to uninsured women, and 12.2% were to women on Medicaid.

**Table 1:** Descriptive Statistics for Total Sample and by Sexual Minority Status

	Total sample (*n* = 5,125)		Sexual minority (*n* = 586)		Not sexual minority (*n* = 4,539)		*p*
	*M*/%	*SE*	*M*/%	*SE*	*M*/%	*SE*	
Prenatal care 1st trimester	92.54		93.78		92.38		.026
No. state-level LGB policies (0 to 3+)	1.14	0.09	1.32	0.13	1.12	0.08	.090
Same-sex marriage	11.37		17.54		10.56		.003
Same-sex adoption	18.23		23.72		17.51		.006
Hate crime statute	55.47		56.90		55.28		.678
Employment discrimination	32.19		38.69		31.34		.040
Race/ethnicity							
Non-Hispanic white	71.72		76.68		71.08		.114
Non-Hispanic black	14.12		9.14		14.76		
Hispanic	10.38		10.37		10.38		
Other race/ethnicity	3.78		3.80		3.78		
Maternal age	27.78	0.16	28.19	0.37	27.72	0.16	.177
Not married/cohabitating w/ preg partner	13.67		16.33		13.32		.207
College graduate, prior to preg	28.57		20.90		29.57		.082
Adolescent poverty	13.35		12.44		13.47		.663
Insurance status, prior to preg							
Not insured	19.48		25.66		18.67		.062
Private Insurance	67.86		60.89		68.77		
Medicaid	12.21		12.51		12.17		
Other	0.46		0.93		0.39		
Birth order							
First	68.80		76.98		67.73		.004
Second	19.04		14.12		19.68		
Third or higher	12.16		8.90		12.59		

Data come from Add Health, Waves I–V.

*SE* standard error.

There were some differences in the state-policy environment prior to pregnancy by sexual minority status. Compared to heterosexual women, births reported by SMW were in states that had slightly more LGB-specific protective policy measures in place (1.30 vs. 1.16, *p* < .09). For example, 23.7% of the births to SMW were in states that allowed same-sex marriage or marriage-like recognition compared to 10.6% of births to heterosexual women (*p* < .0.01), 23.7% of births to SMW were in states that had legalized same-sex adoption compared to 17.5% of heterosexual women (*p* = .01), and 38.7% of births to SMW were in states that prohibited employment discrimination compared to 31.3% of births to heterosexual women (*p* = .04). No statistical differences in other study covariates were found between births to SMW and those to heterosexual women.

### Multivariable Analyses

**Fig. 1.**
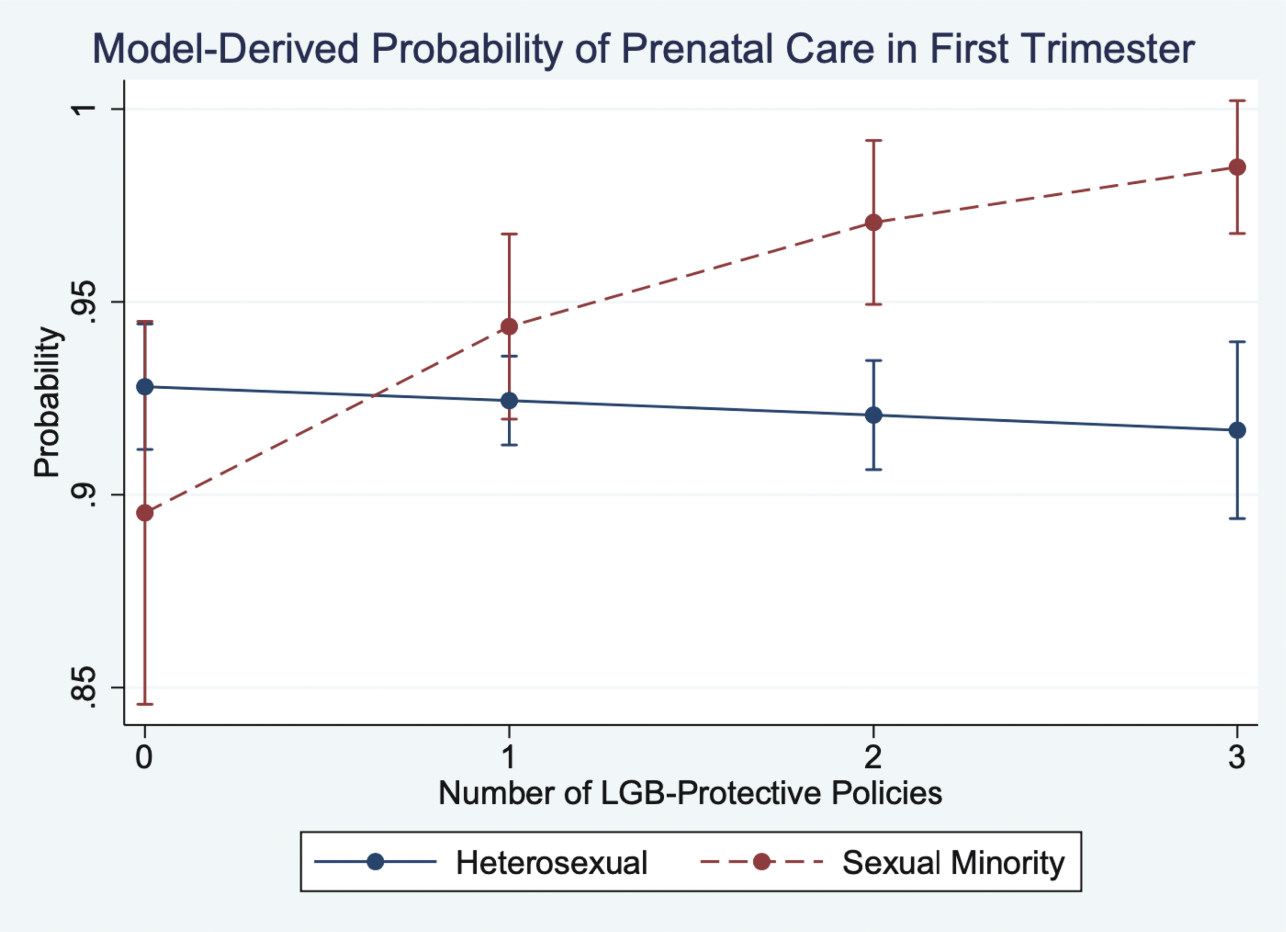
Model-Derived Probability of Prenatal Care in the First Trimester.

In Table 2, we present the results from the multivariable models using the scaled indicator of the number of LGB-specific policies. Model 1 included LGB policies, sexual minority status, and the interaction between the two, and Model 2 included all other covariates. The results showed no direct effect of LGB-specific policies for the reference category: pregnancies to heterosexual women (OR = 1.02, *p* = .48). The interaction between sexual minority status and LGB policies, however, was significant (OR = 2.02, *p* = .01), suggesting that as the number of LGB policies increases, so did the odds of receiving prenatal care in the first trimester for pregnancies reported by SMW. In model 2, we adjusted for all additional covariates. The main results were robust to the inclusion of these additional covariates. The fully adjusted results derived from Model 2 are also presented in Fig. 1.

**Table 2:** Results from Multivariate Models

	Model 1			Model 2		
	OR	95% CI	*p*	OR	95% CI	*p*
Total LGB protections	1.02	(0.35, 1.23)	.745	0.94	(0.82, 1.10)	.480
Sexual minority	0.65	(0.89, 1.18)	.187	0.65	(0.36, 1.19)	.165
LGB protections*sexual minority	2.02	(1.22, 3.35)	.006	2.11	(1.29, 3.44)	.003
Race/ethnicity						
Black				0.61	(0.39, 0.95)	.030
Hispanic				0.56	(0.37, 0.86)	.008
Other				0.67	(0.34, 1.33)	.251
Maternal age				0.99	(0.95, 1.05)	.947
College graduate (prior to pregnancy)				1.99	(1.25, 3.17)	.004
Adolescent poverty				1.05	(0.69, 1.59)	.821
Not married/cohabitating w/ pregnancy partner				0.48	(0.33, 0.67)	.000
Insurance status, prior						
Private insurance				1.41	(0.97, 2.05)	.071
Medicaid				1.25	(0.77, 2.06)	.367
Other				1.01	(0.12, 8.73)	.989
Parity (first birth)						
Second				1.02	(0.64, 1.63)	.934
Third or more				0.67	(0.45, 1.00)	.056

Data come from Add Health, Waves I–V.

*CI* confidence interval; *OR* odds ratio.

We also examined the individual relationship of each policy on prenatal care use (Table 3). Similar to Table 2, Model 1 included the interaction between individual policies and sexual minority status, and Model 2 included all additional covariates. In Table 3, the direct effects of each LGB-specific policy were not significant, suggesting these policies are not associated with prenatal care use among heterosexual women’s pregnancies. The interactions between sexual minority status, same-sex marriage (Panel A), employment discrimination (Panel B), and hate crime statutes (Panel D) were all statistically significant, however, suggesting that the presence of any one of these policies was associated with increased the likelihood of accessing prenatal care in the first trimester for pregnancies reported by SMW. The only policy that was not significantly associated with prenatal care use was same-sex adoption.

**Table 3:** Multivariate Results by Single Policy Indicators

	Model 1			Model 2		
	OR	95% CI	*p*	OR	95% CI	*p*
Panel A: Same-sex marriage						
LGB protection	0.93	(0.58, 1.50)	.869	0.93	(0.56, 1.58)	.791
Sexual minority	1.04	(0.63, 1.73)	.761	1.09	(0.67, 1.77)	.736
LGB protection × sexual minority	*6.46*	*(1.08, 38.72)*	*.041*	*6.79*	*(1.15, 41.35)*	*.038*
Panel B: Employment discrimination						
LGB protection	1.08	(0.77, 1.51)	.667	0.89	(0.63, 1.28)	.556
Sexual minority	0.84	(0.49, 1.45)	.528	0.86	(0.51, 1.44)	.561
LGB protection × sexual minority	*5.74*	*(1.78, 18.56)*	*.003*	*6.78*	*(2.09, 21.97)*	*.001*
Panel C: Same-sex adoption						
LGB protection	1.47	(0.96, 2.24)	.073	1.12	(0.72, 1.73)	.616
Sexual minority	1.11	(0.66, 1.88)	.689	1.14	(0.69, 1.89)	.620
LGB protection × sexual minority	1.87	(0.43, 8.25)	.407	2.3	(0.51, 10.26)	.276
Panel D: Hate crime statute						
LGB protection	0.93	(0.67, 1.29)	.673	0.85	(0.60, 1.22)	.387
Sexual minority	0.73	(0.38, 1.41)	.357	0.78	(0.42, 1.44)	.422
LGB protection × sexual minority	*3.11*	*(1.17, 8.31)*	*.023*	*3.08*	*(1.16, 8.18)*	*.024*

Data come from Add Health, Waves I–V; Model 2 adjusts for race/ethnicity, maternal age, education, adolescent poverty, relationship status, insurance status, and birth order.

*CI* confidence interval; *OR* odds ratio; *p* probability value.

### Sensitivity Analyses

While we time-ordered are exposure variable such that we use the policy indicators that precede the birth because the policies capture the state-level environment at Wave III and IV, we cannot be certain that the policies in place at that time reflect the policy environment at the exact time of the birth. Thus, we conducted two supplementary analyses. The first uses a cumulative exposure variable that sums up Waves III and IV policy environment scores to capture “midlife” total exposure. Second, we restricted our analyses to individuals who did not move more than 50 miles from their original global positioning system-located home location between Waves III and IV. As stated before, Add Health does not provide the state in which a respondent lives, however, they do release information on distance between home location between waves of data collection. These results are provided in Table 4.

**Table 4:** Sensitivity Analyses for LGB Policy and Prenatal Care

	Model 1			Model 2		
	OR	95% CI	*p*	OR	95% CI	*p*
Panel A: Cumulative exposure (*n* = 4,808)						
LGB protections	1.04	(0.39, 1.49)	.434	1.01	(0.92, 1.10)	.832
Sexual minority	0.76	(0.39, 1.49)	.346	0.69	(0.36, 1.33)	.269
LGB protection × sexual minority	*1.28*	*(1.02, 1.61)*	*.034*	*1.35*	*(1.07, 1.70)*	*.011*
Panel B: Non-movers (*n* = 3,266)						
LGB protections	1.05	(0.89, 1.23)	.547	0.99	(0.83, 1.17)	.881
Sexual minority	0.62	(0.30, 1.32)	.217	0.6	(0.30, 1.19)	.145
LGB protection × sexual minority	*2.2*	*(1.19, 4.06)*	*.012*	*2.26*	*(1.28, 4.36)*	*.006*

Data come from Add Health, Waves I–V; Model 2 adjusts for race/ethnicity, maternal age, education, adolescent poverty, relationship status, insurance status, and birth order.

*CI* confidence interval; *OR* odds ratio; *p* probability value.

Panel A of Table 4 shows that, like Table 2, there is no statistically significant relationship between the cumulative policy environment for the referent category, births to heterosexual women (OR = 1.04, *p* = .43). The interaction between sexual minority identity and cumulative policy environment was statistically significant shows that as the number of LGB-protective policies increases, so do the odds of receiving prenatal care in the first trimester for pregnancies reported by SMW (OR = 1.28, *p* = .03). These results were robust to the inclusion of all control variables in Model 2.

Panel B presents the results for the individuals who did not move between Waves III and IV of the study. This set of supplementary analyses uses the policy count prior to birth. The results are also like those presented in Table 2 in both Model 1 and the fully adjusted Model 2: There was no statistically significant relationship between LGB policy protections and prenatal care use for pregnancies reported by heterosexual women (Model 2 OR = 0.99, *p* = .88). The interaction between sexual minority identity and LGB policies showed that increases in the number of LGB-protective policies prior to birth was associated with an increased odds in prenatal care in the first trimester for pregnancies reported by SMW (Model 2 OR = 2.26, *p* = .01).

## Discussion

These results are the first to examine sexual orientation and prenatal care use in the first trimester, as well as incorporate the role of *modifiable* social policies on prenatal care use in the first trimester. Critically, our results suggest that the use of prenatal care in the first trimester for SMW was closely tied to the social and political landscape, such that SMW in states without legal protections are significantly less likely to use prenatal care compared to heterosexual-identified women. In states with multiple forms of legal protections, however, SMW were *more* likely to use prenatal care in the first trimester than their heterosexual peers. These results reflect those of Hatzenbuehler et al. [29], which showed legal protections for sexual minorities were associated with greater preventative care use services among gay men. Our results extend these findings to SMW and pregnancy; social policies that confer legal protections to SMW were associated with increases in the use of prenatal care in the first trimester. While we cannot determine the exact pathways through which these policies operate, there are likely multiple mechanisms, including higher levels of trust in medical institutions, decreased fear of discrimination, and higher rates of self-efficacy that translate into more health-improving behaviors.

First, these policies may influence healthcare-seeking behavior by reducing the stigma that SMW feel, especially during a vulnerable period. Research shows that fear of stigma/discrimination and the cost of care are consistent barriers to prenatal care, especially among minoritized populations [47]. Living in states that signal SMWs’ value and their families’ value to society by formally recognizing their unions and criminalizing discrimination are important signals to LGBTQ families that they are not just tolerated but accepted and supported by their communities. Indeed, research has shown that pregnancy among queer women comes with unique concerns and considerations, including whether their pregnancy will be celebrated by family members and providers or met with hostility, either outright or through a series of microaggressions [19, 25, 27, 48]. These concerns may lead to delayed prenatal care use among SMW as a strategy to avoid potentially harmful interactions. Relatedly, other research has shown that structural LGBTQ stigma is associated with mental health and self-efficacy [28], which may increase the likelihood that pregnant SMW in states with more protective policies engage in self-efficacious behaviors, including prenatal care use.

Second, these policies may increase insurance coverage and stability in family formation and provide legal protections that create economic stability for queer families and individuals, increasing their ability to access and willingness to use prenatal care services. Historically, same-sex sexual orientation was considered a mental illness, and research has found long-term impacts on trust in medical institutions among sexual minorities. Negative experiences in healthcare settings often reinforce these beliefs [49]. Policies that explicitly state that individuals may not be discriminated against because of their sexual orientation may improve trust in social institutions and facilitate health care utilization. Other research has shown that policies have been shown that changes in state-level policies providing additional protections for immigrants increase prenatal care use among migrant populations [50]. Protections conferred by the state-level policy indicators in our scale likely result in increases in financial and legal security that facilitate the use of preventative care services.

Third, these policies may be indicative of other structural forms of stigma and discrimination, such as structural sexism, as articulated by Everett et al. [32] in their paper on structural heteropatriarchy, which documented high levels of correlation between structural sexism and LGB policy environments. Additionally, the states with the poorest LGB policy classifications, according to the Movement Advancement Project (e.g., TX, FL, SC, AL) [51], also did not expand Medicaid and had some of the lowest levels of TANF benefits for individuals in their state [52]. It is not surprising that these states also have some of the highest rates of infant and maternal mortality. Notably, the LGB policies’ *specific* relationship to prenatal care use among births SMW, and not births to heterosexual women, suggests that while states with more LGB policies in place may be safer places for pregnant people in general, there is a unique benefit for pregnant SMW prenatal care use associated with more sexual orientation-based protections.

### Limitations

Our study has several limitations that should be noted. First, we combined all sexual minority identities into a single category due to limited statistical power. While supplementary analyses showed similar relationships across each identity group, future research should replicate these results and explore nuances within the sexual minority population to the extent possible. Second, we cannot determine whether sexual orientation is moderated by other sociodemographic characteristics such as race, ethnicity, or socioeconomic status due to sample size limitations. Future research should examine whether these findings differ in more diverse samples of SMW. Third, our results focus on when prenatal care was initiated. We do not have indicators of quality or satisfaction with prenatal care, which may be more important correlates of maternal and infant health outcomes. Fourth, we do not have access to information regarding *which* state the respondent resided in during data collection. Future research with this type of information could incorporate multilevel models that account for shared variance across pregnancies within states. Fifth, just 6% of the sample did not report receiving prenatal care in the first trimester. Overall, this is good news; the overwhelming majority of pregnant women are seeking prenatal care during the recommended time frame. Shifts in the policies that create more inclusive environments may be an important pathway to ensuring *all* women receive prenatal care in the first trimester.

Finally, our sample is limited to cisgender women. Gender identity was asked in Wave V of Add Health; however, only five births were to respondents who did not identify as “female”; thus, they were removed due to small sample sizes. Of these pregnancies, 1 of the 5 did not report prenatal care in the first trimester; this pregnancy occurred in a state with one policy protection in place. The additional four pregnancies were spread across policy environments, with one residing in a state with zero protections and the other three in states with 2 and 3 protections in place. More work is needed to understand how sexual and gender identities function together to shape prenatal care use.

Barriers to adequate prenatal care may be one contributing factor to the growing body of evidence documenting sexual orientation disparities in maternal, infant, and child health, particularly in states without protections for sexual minority populations. As many qualitative studies have documented, the lack of protective policies may trickle down to negative interpersonal experiences with medical providers. Our findings suggest that policy shifts that reduce stigma and increase legal protections and financial stability may be critical for reducing perinatal health inequities. Moreover, many states still maintain discriminatory laws that may or may not protect LGBTQ people from being denied medical services. For SMW in states where inclusive policies are absent, developing inclusive coordinated care strategies to support SMW may be a critical avenue for future public health interventions, including telehealth and doulas. Such alternative forms of care may allow SMW to circumnavigate hostile medical environments to improve obstetrical and reproductive health.

## Data Availability

De-identified data are available through the application process outlined at https://addhealth.cpc.unc.edu/data/#restricted-use. The analytic code is unavailable to the public as it resides on a restricted access server. The materials of the study are not publicly available.
